# A machine learning‐based survival prediction model of high grade glioma by integration of clinical and dose‐volume histogram parameters

**DOI:** 10.1002/cam4.3838

**Published:** 2021-03-24

**Authors:** Haiyan Chen, Chao Li, Lin Zheng, Wei Lu, Yanlin Li, Qichun Wei

**Affiliations:** ^1^ Department of Radiation Oncology Key Laboratory of Cancer Prevention and Intervention Ministry of Education The Second Affiliated Hospital Zhejiang University School of Medicine Hangzhou Zhejiang China; ^2^ Zhejiang University Cancer Center Hangzhou Zhejiang China; ^3^ Department of Radiation Oncology Taizhou Tumor Hospital Taizhou Zhejiang China; ^4^ Department of Colorectal Surgery and Oncology Key Laboratory of Cancer Prevention and Intervention Ministry of Education The Second Affiliated Hospital Zhejiang University School of Medicine Hangzhou Zhejiang China; ^5^ College of Science Hangzhou Normal University Hangzhou Zhejiang China

**Keywords:** DVH features, high‐grade glioma, machine learning, random survival forest, survival prediction

## Abstract

**Purpose:**

Glioma is the most common type of primary brain tumor in adults, and it causes significant morbidity and mortality, especially in high‐grade glioma (HGG) patients. The accurate prognostic prediction of HGG is vital and helpful for clinicians when developing therapeutic strategies. Therefore, we propose a machine learning‐based survival prediction model by analyzing clinical and dose‐volume histogram (DVH) parameters, to improve the performance of the risk model in HGG patients.

**Methods:**

Eight clinical variables and 39 DVH parameters were extracted for each patient, who received radiotherapy for HGG with active follow‐up. Ninety‐five patients were randomly divided into training and testing cohorts, and we employed random survival forest (RSF), support vector machine (SVM), and Cox proportional hazards (CPHs) models to predict survival. Calibration plots, concordance indexes, and decision curve analyses were used to evaluate the calibration, discrimination, and clinical utility of these three models.

**Results:**

The RSF model showed the best performance among the three models, with concordance indexes of 0.824 and 0.847 in the training and testing sets, respectively, followed by the SVM (0.792/0.823) and CPH (0.821/0.811) models. Specifically, in the RSF model, we identified age, gross tumor volume (GTV), grade, Karnofsky performance status (KPS), isocitrate dehydrogenase (IDH), and D99 as important variables associated with survival. The AUCs of the testing set were 92.4%, 87.7%, and 84.0% for 1‐, 2‐, and 3‐year survival, respectively. According to this model, HGG patients can be divided into high‐ and low‐risk groups.

**Conclusion:**

The machine learning‐based RSF model integrating both clinical and DVH variables is an improved and useful tool for predicting the survival of HGG patients.

## Graphical abstract

We propose the machine learning‐based RSF and SVM models, and the classical CPH model, to identify predictors for survival and examine treatment outcomes in patients with HGG by integrating clinical and DVH parameters.The RSF model showed the best performance among the three models and is an improved and useful tool for the survival prediction of HGG.

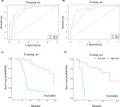

## 1 INTRODUCTION

Glioma is the most common type of primary brain tumor in adults, representing more than 80% of malignant intracranial tumors.^1^, ^2^ The World Health Organization (WHO) pathologically classifies glioma into Grades I, II, III, and IV glioma according to histological features.^3^ Among them, Grades III and IV are categorized as high‐grade glioma (HGG), characterized by poorly differentiated and highly aggressive tumor cells and a poor prognosis.^3^ Despite the standard treatment consisting of surgery followed by radiotherapy and temozolomide (TMZ) chemotherapy,^4^, ^5^ the morbidity and mortality of HGG are still very high.^1^ It is therefore essential to accurately predict the prognosis of patients, to guide clinicians in making personalized treatment decisions and surveillance strategies for patients with different risk levels.

Researchers have made great efforts to understand individual risk profiles and develop survival prediction models. Previous studies have shown that tumor grade, age, Karnofsky performance status (KPS), extent of surgery, and other clinical data are important prognostic factors for HGG patients.^6^ A few molecular markers, such as isocitrate dehydrogenase (IDH) 1 and 2 mutations, 1p/19q chromosomal codeletion, and *O*‐6‐methylguanine‐DNA methyltransferase (MGMT) promoter methylation, have also been suggested to be useful in survival prediction.^7^, ^8^ Interestingly, recent studies have elucidated that dosimetric parameters can affect prognosis. For example, Yang et al. found that the dose‐volume histogram (DVH) signature reflecting the planning score was an independent predictor of progression‐free survival in locoregionally advanced nasopharyngeal carcinoma.^9^ Rijkmans et al. evaluated the association of dosimetric parameters with tumor regression in local advanced rectal cancer and found that tumor volume is the most important predictive factor.^10^ Considering the crucial role of radiotherapy in the treatment course, in this study, we will integrate clinical variables with DVH parameters to predict the overall survival (OS) of HGG patients.

To resolve this issue, different approaches can be used. The classical approach—the Cox proportional hazard (CPH) model—can be employed to identify clinical parameters and dosimetric parameters that significantly affect the outcome of interest. The CPH model is the most frequently used method in survival analysis because of its convenience. However, it assumes that the outcome is a linear combination of covariates, but patient data are diverse and complex and generally, they cannot be considered linear.^11^, ^12^ The random survival forest (RSF) model, one of the most widely used methods of machine learning, enables the detection of relationships from complex datasets. RSF is a flexible nonparametric tree‐ensemble method for the analysis of right‐censored survival data.^13^ It builds hundreds of trees and outputs the results by voting.^14^ In addition, it reduces variance and bias by using all variables collected and by automatically assessing nonlinear effects and complex interactions.^12^ In addition to RSF, the support vector machine (SVM) is another supervised machine learning algorithm used for classification and regression.^15^ It separates the data by constructing a hyperplane in a high‐ or infinite‐dimensional space, and it can classify nonlinear data using the kernel trick.^16^

Therefore, in this study, we propose three models, namely, the machine learning‐based RSF and SVM models, and the classical CPH model, to identify predictors of survival and examine treatment outcomes in patients with HGG by integrating clinical and DVH parameters. In addition, we used the calibration plot, concordance index (c‐index), and decision curve analyses to evaluate the calibration, discrimination, and clinical utility of these three models.

## 2 METHODS AND MATERIALS

### 2.1 Patients and treatment

Patients diagnosed with HGG at the Second Affiliated Hospital, Zhejiang University School of Medicine, from January 2015 to June 2018 were consecutively enrolled in this study. All patients underwent surgery and were histologically diagnosed with WHO Grades III–IV glioma. In addition, patients underwent postoperative intensity‐modulated radiation therapy (IMRT) with standard contouring, with concomitant TMZ chemotherapy. Those who did not receive IMRT with standard contouring or without follow‐up were excluded. Clinical data on patient age, gender, grade, KPS, cycles of adjuvant chemotherapy, MGMT expression, IDH1 R132 mutation, Ki‐67 expression, and OS were collected. Specifically, MGMT expression, IDH1 R132 mutation, and Ki‐67 expression were determined by immunohistochemistry in postoperative tissues. OS was defined as the number of months between the date of diagnosis and the date of death from any cause. For concurrent chemotherapy, TMZ was administered once daily (7 days/week) at a dose of 75 mg/m^2^/day. For adjuvant chemotherapy, TMZ started 4 weeks after the completion of radiotherapy. Adjuvant TMZ was administered at a dose of 150 mg/m^2^ during the first cycle (Days 1–5 per 28‐day cycle) if no toxicity was observed. Then, TMZ was administered at a dose of 200 mg/m^2^ from the second cycle onwards.^17^ This project was approved by the Independent Ethics Committee of the Second Affiliated Hospital, Zhejiang University School of Medicine, and informed consent was obtained from all patients.

### 2.2 IMRT planning and DVH feature extraction

All patients were immobilized in the supine position by a thermoplastic head fixation mask, followed by CT simulation. The generated CT images were fused with enhancement magnetic resonance imaging (MRI) images and delineated slice‐by‐slice. According to our protocol, gross tumor volume (GTV) was defined as the surgical cavity and any residual contrast‐enhancing tumor on postcontrast T1‐weighted MRI, ignoring any edematous region.^18^ Clinical target volume (CTV) 1 was GTV plus a margin of 1 cm, while CTV2 was GTV plus a margin of 2 cm. In addition, both were modified to avoid barriers of spread (i.e., bone, falx cerebri, ventricles). Then, the corresponding planning target volume (PTV) 1 and PTV2 were obtained by expanding the CTVs by 3 mm. The standard fractionation scheme was a dose of 60 Gy delivered in 30 fractions (2 Gy per day from Monday to Friday, 6 weeks). The prescription dose was 60 Gy for CTV1 and 54 Gy for CTV2 by the use of the Eclipse 10.0 treatment planning system.^19^ A 6‐MV photon beam was provided by the Varian Trilogy LINAC with a multileaf collimator.

DVH features were collected from the initial treatment plans, including GTV, CTV1, CTV2, equivalent spherical diameter, minimal dose, maximal dose, mean dose, modal dose, STD, EUD, TCP, the dose that covered 1% to 99% of the CTV2 (D1–D99), and the percent volume of CTV2 that received the dose of 50 Gy to 65 Gy (V50–V65). Some of the DVH features for each patient are exported directly from DVH text files, and the rest of the features such as modal dose, STD, EUD, TCP, D1–D99 and V50–V65 of CTV2, were calculated from the DVH with a program written in MATLAB (MATLAB R2011b, Mathworks, Inc.). The modal dose is the most frequent dose in CTV2. STD is the standard deviation of the dose distributions in CTV2. EUD represents the equivalent uniform dose which leads to the same control probability as the nonuniform dose distribution.^20^ Therefore, it can be used to compare the local control or radiobiological effect for different dose distributions. TCP is the EUD‐based tumor control probability.^21^ The EUD and TCP equations are as follows:

$$\mathrm{EUD}={\left(\sum_{i}v_{i}D_{i}^{a}\right)}^{\frac{1}{a}}$$

$$\mathrm{TCP}=\frac{1}{1+{\left(\frac{D_{50}}{\mathrm{EUD}}\right)}^{4\gamma_{50}}}$$

where a is a specific parameter of the EUD model, the D_i_ and v_i_ data pairs are obtained from the differential DVH, the TCD_50_ is the dose to achieve 50% tumor control, and γ_50_ is a specific parameter to describe the slope of the dose response curve. In this study, TCD_50_ and γ_50_ were set to −10, 60, and 2, respectively.^20^, ^22^, ^23^ All doses are described as the total dose per course.

### 2.3 RSF model

To make the statistics easier to analyze, we transformed all the continuous variables into categorical variables by the maxstat package using R version 3.6.1 (R Foundation for Statistical Computing), which is a bioinformatics tool to determine the optimal cut point for one or multiple continuous variables. The included patients were randomly divided into training and testing cohorts. The data were sampled using random bootstrapping to generate a training dataset. To minimize classification error in the training data, the model randomly selected a subset of feature variables (m_try_) to obtain the optimal result. The corresponding trees of the training set were repeatedly generated until the out‐of‐bag (OOB) error rate had stabilized. Then the RSF model selected the model with the lowest OOB error rate. On the basis of the RSF model, the predicted survival time and risk score of each patient was calculated. The optimal cutoff point of the corresponding patient risk scores with the smallest Kaplan–Meier log‐rank was determined by the maxstat package in R software. We used the cutoff point to stratify patients into high‐ and low‐risk groups. Then, Kaplan–Meier survival curves of the high‐ and low‐risk groups were generated using the survminer R package.

### 2.4 SVM model

The SVM model was constructed based on the training dataset via the caret package in R software. Each included feature was ranked according to its predictive ability. To obtain the optimal number of features to build the model, a 5‐fold cross‐validation approach was applied in the training set, and the variable number was identified according to the cross‐validation accuracy. The established model was then used to predict the samples in the testing dataset.

### 2.5 CPH model

In the CPH model, univariate and multivariate models were constructed to evaluate factors correlated with survival. Univariate and multivariate Cox regression analyses were conducted to generate hazard ratios (HRs) with confidence intervals (CIs). Then, we calculated the predicted 1‐, 2‐, and 3‐year survival probabilities for each patient using the nomogram that was constructed on the basis of the CPH model.

### 2.6 Calculation of the area under the ROC curve

Receiving operator characteristic (ROC) curves were used to assess the predictive accuracy of the different survival analysis models (CPH, SVM, and RSF models). The area under the ROC curve (AUC) was used as a performance metric for all the models. The ROC curve and AUC were obtained by using the timeROC package in R. The AUC was calculated to compare the discriminatory ability of the above models.

### 2.7 Construction and evaluation of the nomogram model

The nomogram of each model was constructed via the rms package in R based on the selected features in the RSF, SVM, and CPH models. Calibration plots were constructed and the c‐indexes were calculated to evaluate the predictive accuracy of the nomogram model. DCA, which compares benefit versus harm, was used to estimate whether the clinical utility of the prediction models would do more harm than good. The net benefits were quantified to determine the clinical utility of each model.

### 2.8 Statistics

The Pearson *χ* ^2^ test was employed to investigate significant differences between the training set and testing set. DVH features were exported from the treatment planning system and calculated by MATLAB software. All statistical analyses and plotting were conducted using R version 3.6.1. A *p* value of <0.05 was considered statistically significant. All tests were two sided, and 95% CIs were used.

## 3 RESULTS

### 3.1 Clinical and pathological characteristics of the included patients

**FIGURE 1:**
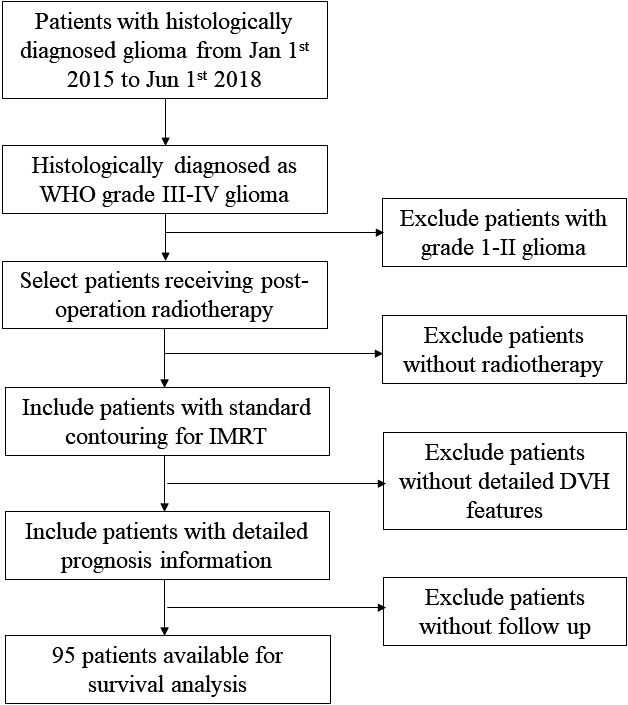
Flow chart of patient inclusion

A total of 95 patients were assessed as eligible for inclusion in this study by using the patient selection algorithm described in the Methods section (Figure 1). The clinicopathological characteristics of these 95 patients are shown in Table 1. There were 26.3% patients (*N* = 25) with Grade III glioma and 73.7% patients (*N* = 70) with Grade IV glioma. Among them, 63.2% (*N* = 60) were younger than 60 years, and 36.5% (*N* = 35) were older than 60 years. Gender was almost evenly distributed, with 51.6% male patients and 48.4% female patients. In terms of the patients’ functional abilities, which were evaluated by the KPS in this study, most (*N* = 84, 88.4%) had good functional status, with KPS scores of more than 60.

**TABLE 1:** Clinical and pathological characteristics of included patients

	Total patients	Training set	Testing set	*p* value
Total patients	95 (100%)	57 (60.0%)	38 (40.0%)	
Age				0.385
≤60	60 (63.2%)	34 (59.6%)	26 (68.4%)	
>60	35 (36.8%)	23 (40.4%)	12 (31.6%)	
Gender				0.054
Male	49 (51.6%)	34 (59.6%)	15 (39.5%)	
Female	46 (48.4%)	23 (40.4%)	23 (60.5%)	
Grade				0.634
III	25 (26.3%)	14 (24.6%)	11 (28.9%)	
IV	70 (73.7%)	43 (75.4%)	27 (71.1%)	
KPS				0.793
≤60	11 (11.6%)	7 (12.3%)	4 (10.5%)	
>60	84 (88.4%)	50 (87.7%)	34 (89.5%)	
Adjuvant chemotherapy				0.324
<6 cycles	41 (43.2%)	28 (49.1%)	13 (34.2%)	
6 cycles	31 (32.6%)	19 (33.3%)	12 (31.6%)	
>6 cycle	23 (24.2%)	10 (17.5%)	13 (34.2%)	
MGMT expression				0.08
Negative	78 (82.1%)	50 (87.7%)	28 (73.7%)	
Positive	17 (17.9%)	7 (12.3%)	10 (26.3%)	
IDH1 R132H mutation				0.452
WT	69 (72.6%)	43 (75.4%)	26 (68.4%)	
MUT	26 (27.4%)	14 (24.6%)	12 (31.6%)	
Ki−67				0.587
≤50	65 (68.4%)	39 (68.4%)	26 (68.4%)	
>50	30 (31.6%)	18 (31.6%)	12 (31.6%)	
Meadian OS	38.30 (31.60–45.00)	44.24 (34.19–54.29)	30.81 (26.23–35.39)	0.113

Twenty‐four percent of patients (*N* = 23) did not receive any adjuvant chemotherapy, 51.6% of patients (*N* = 49) received less than 6 cycles of adjuvant chemotherapy, and the remaining patients (*N* = 23, 24.2%) received more than 6 cycles of adjuvant chemotherapy. We also included pathological factors, such as MGMT expression, IDH1 R132H mutation, and Ki‐67 expression. Specifically, MGMT expression in 82.1% of patients (*N* = 78) was negative, and most of the patients (*N* = 78, 82.1%) did not carry the IDH1 R132H mutation. Moreover, Ki‐67 expression in 68.4% of patients (*N* = 65) was greater than 50%. The median OS was 38.3 months, ranging from 31.6 to 45.0 months.

### 3.2 Characteristics of DVH features

**FIGURE 2:**
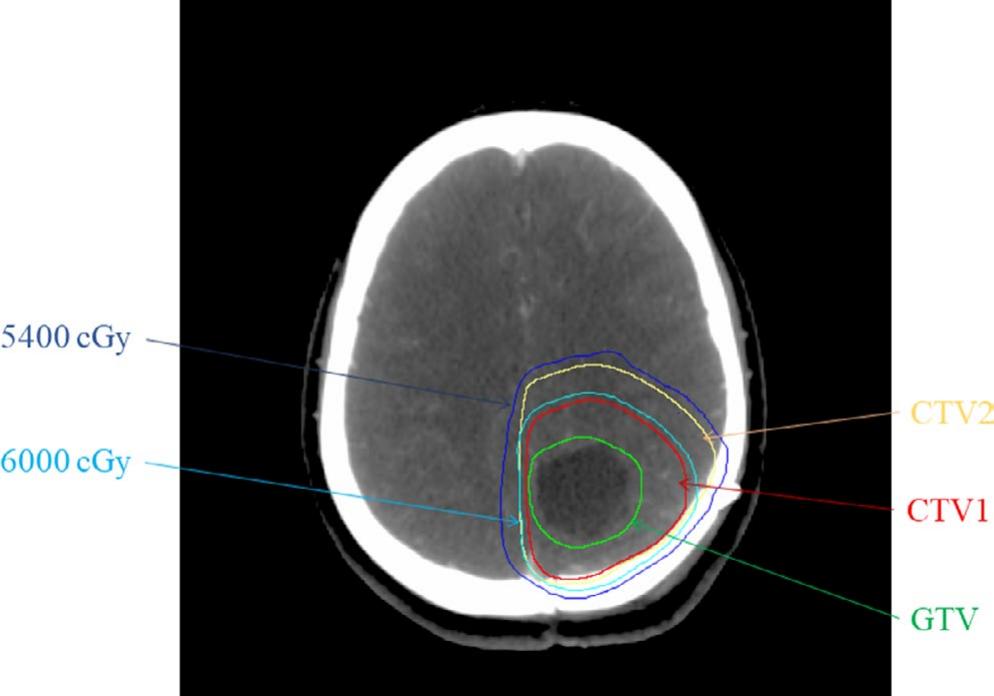
An example of treatment planning in a patient with HGG

For patients with HGG, the NCCN guidelines recommended surgery first, followed by radiotherapy and concurrent chemotherapy, and then adjuvant chemotherapy. The contouring and treatment planning are specifically described in the Methods and Materials section. Figure 2 depicts the contoured structures and dose planning. In this study, we extracted 39 DVH features from the initial treatment plans, including GTV, CTV1, CTV2, equivalent spherical diameter, minimal dose, maximal dose, mean dose, modal dose, STD, TCP, EUD, D1–D99, and V50–V65 of CTV2 (Table S1). Previous research illustrated the effects of DVH parameters on the biological outcomes of patients. To explore the association between DVH features and prognosis, we transformed these continuous variables into categorical variables to simplify the following statistical analyses (Table S1).

### 3.3 Machine learning‐based RSF model for predicting prognosis

**FIGURE 3:**
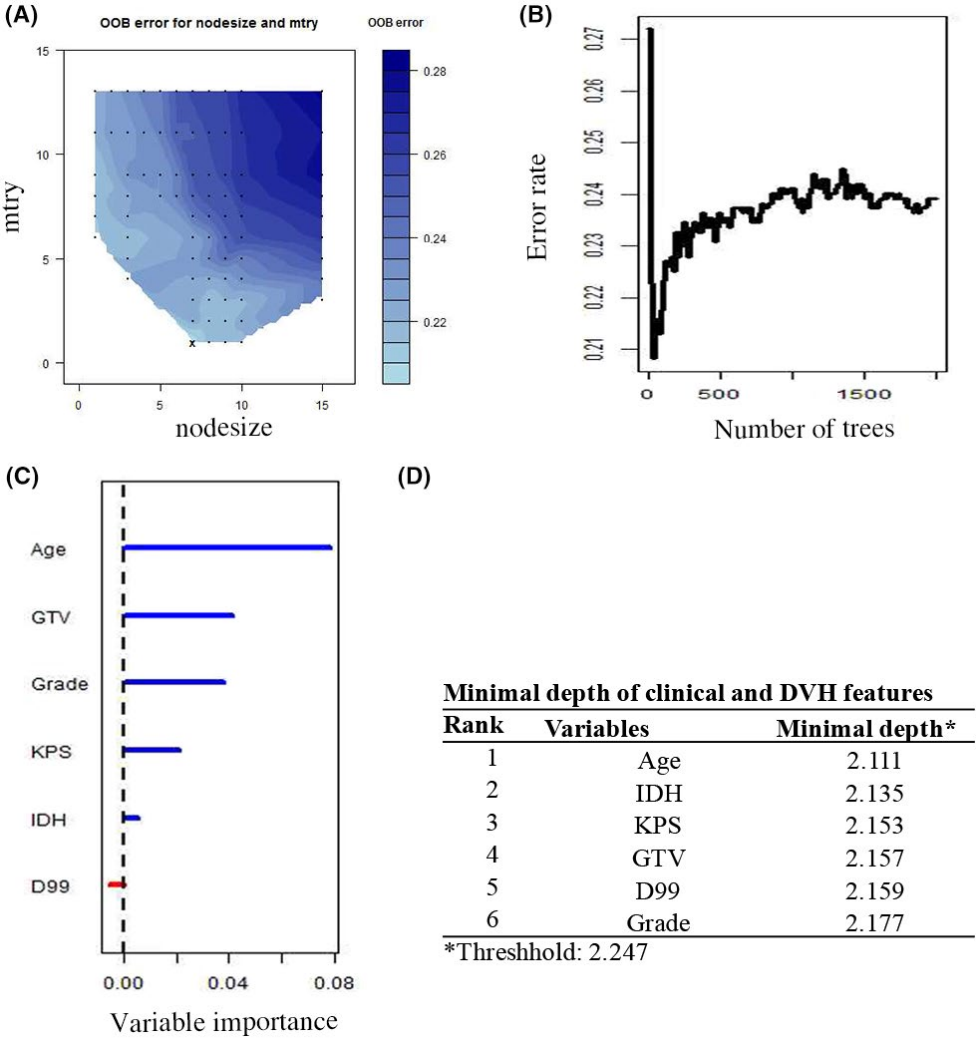
(A and B) Out‐of‐bag (OOB) error rate to assess the quality of the survival prediction for HGG in the RSF model. The OOB error rate was 21.11% with mtry = 1, nodesize = 7 (A), and ntree value = 2000 (B). (C and D) Minimal depth to predict the survival of HGG patients in the RSF model. We identified six significant factors associated with survival, whose minimal depth was lower than the threshold of 2.247. Age was of the highest importance, followed by GTV, grade, KPS, IDH, and D99

The random forest method is a machine learning technique used for classification and regression, presenting several advantages over other predictive methods. Therefore, we used random forest to predict the prognostic factors of HGG patients. First, the entire dataset was split into two mutually exclusive datasets, with 60% assigned to the training set (*N* = 57) and 40% assigned to the testing set (*N* = 38). There were no statistically significant differences in terms of clinicopathological features or survival outcomes between the two sets (Table 1). Then, we obtained the optimal results with an m_try_ value of 1, a nodesize value of 7, and an ntree value of 2000, which yielded a low OOB error rate of 21.11% in the training set (Figure 3A and B). Finally, we identified six significant factors strongly associated with survival, whose minimal depth was lower than the threshold of 2.247. Of those, age was of the highest importance, followed by GTV, grade, KPS, IDH, and D99 (Figure 3C and D).

**FIGURE 4:**
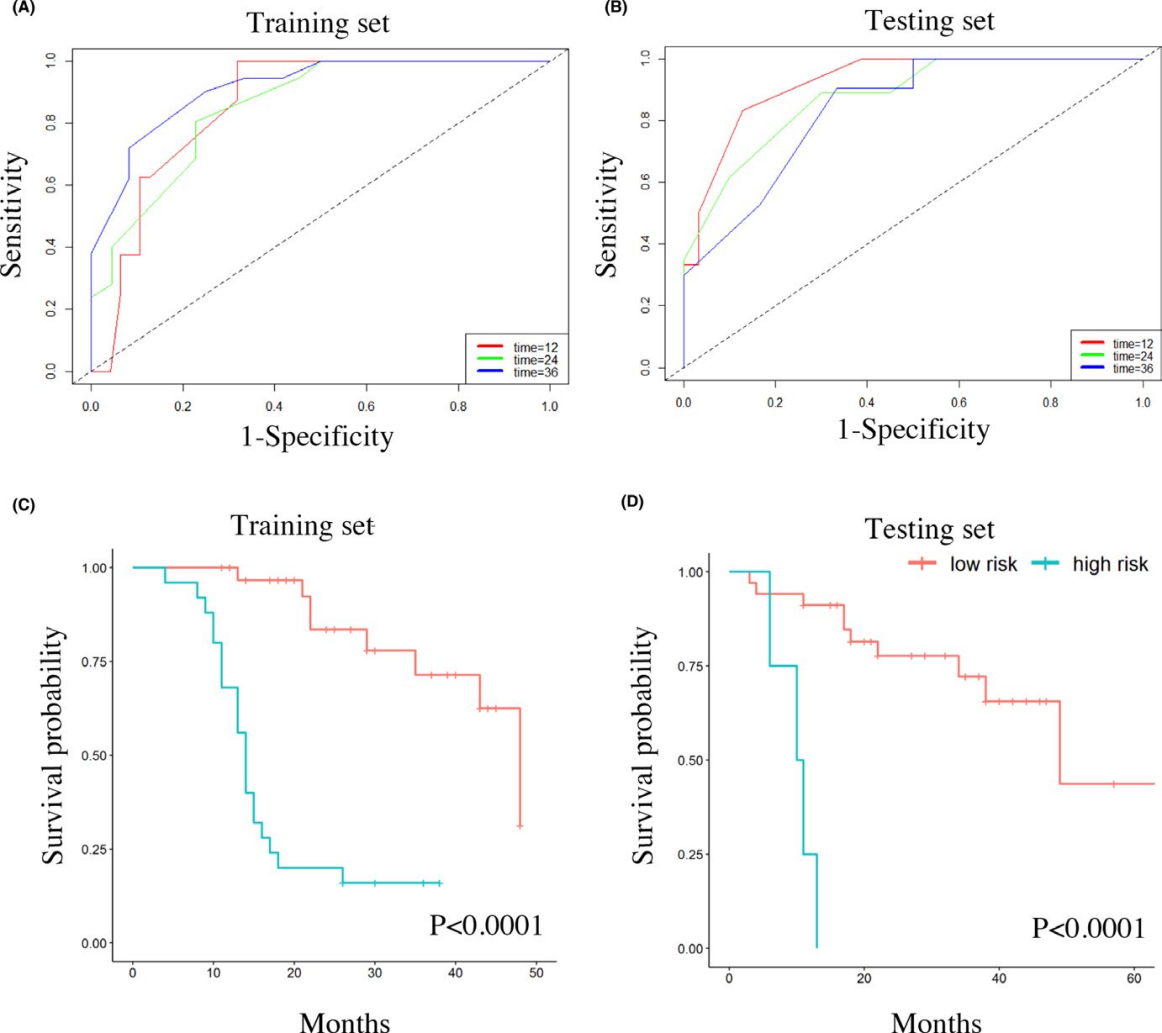
(A) The ROC curves of the training set in the RSF model. The AUCs were 85.6% (95% CI [75.63%, 95.65%]), 85.4% (95% CI [74.81%, 96.08%]), and 91.4% (95% CI [81.92%, 99.99%]) for 1‐, 2‐, and 3‐year survival, respectively. (B) The ROC curves showed that the AUCs were 92.4% (95% CI [83.63%, 99.99%]), 87.7% (95% CI [75.65%, 99.78%]), and 84.0% (95% CI [68.82%, 99.14%]) for 1‐, 2‐, and 3‐year survival in the testing set, respectively. (C and D) HGG patients were divided into high‐ and low‐risk groups according to the RSF model. The low‐risk group had a longer OS time than the high‐risk group in both the training (HR = 9.075, 95% CI [3.603, 22.86], *p* < 0.0001) (C) and testing sets (HR = 17.4394, 95% CI [3.738, 81.37], *p* < 0.0001) (D)

Based on these variables, we generate our RSF model. Figure 4A and B shows the ROC curves of the training and testing sets at 1, 2, and 3 years. For 1‐, 2‐, and 3‐year survival, the AUCs were 85.6% (95% CI [75.63%, 95.65%]), 85.4% (95% CI [74.81%, 96.08%]) and 91.4% (95% CI [81.92%, 99.99%]), respectively, in the training set and 92.4% (95% CI [83.63%, 99.99%]), 87.7% (95% CI [75.65%, 99.78%]) and 84.0% (95% CI [68.82%, 99.14%]), respectively, in the testing set (Table 2). Based on the RSF model, we divided the patients into high‐ and low‐risk groups (Figure 4C and D). The low‐risk group had a longer OS than the high‐risk group in both the training (HR = 9.075, 95% CI [3.603, 22.86], *p* < 0.0001) and testing sets (HR = 17.4394, 95% CI = 3.738–81.37, *p* < 0.0001). Overall, this machine‐learning‐based survival prediction model enables us to identify HGG patients who are at risk of poor outcomes.

**TABLE 2:** AUCs of 1‐year, 2‐year and 3‐year survival in RSF, SVM and CPH models

		ACU (95% CI)		
		1‐year survival	2‐year survival	3‐year survival
Machine learning‐based RSF model	Training set	85.6% [75.63%, 95.65%]	85.4% [74.81%, 96.08%]	91.4% [81.92%, 99.99%]
	Testing set	92.4% [83.63%, 99.99%]	87.7% [75.65%, 99.78%]	84.0% [68.82%, 99.14%]
Machine learning‐based SVM model	Training set	82.6% [71.95%, 93.21%]	83.3% [71.88%, 94.73%]	88.5% [77.51%, 99.53%]
	Testing set	89.5% [76.93%, 99.99%]	87.1% [76.25%, 97.98%]	82.3% [67.38%, 97.39%]
Classical CPH model	Training set	83.5% [72.36%, 94.66%]	87.1% [76.96%, 97.24%]	91.6% [82.05%, 99.99%]
	Testing set	87.9% [74.01%, 99.99%]	87.3% [76.04%, 98.68%]	84.9% [68.93%, 99.99%]

### 3.4 Machine learning‐based SVM model for predicting prognosis

**FIGURE 5:**
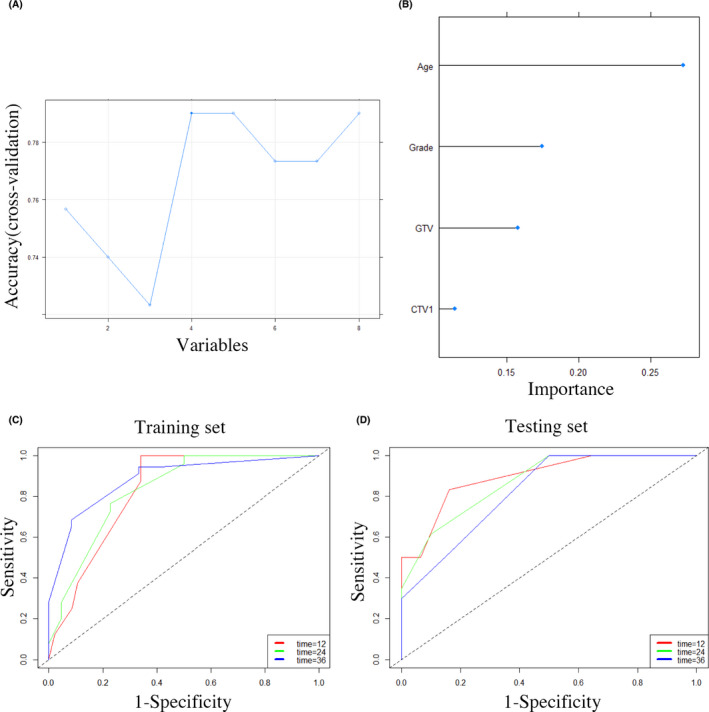
(A) The prediction accuracy with a certain number of features in the SVM model. When the number of features was four, it achieved the optimal accuracy of 77%. (B) The top four features included in the SVM model were age, grade, GTV and CTV1. (C and D) In the SVM model, the AUCs for 1‐, 2‐, and 3‐year survival were 82.6% (95% CI [71.95%, 93.21%]), 83.3% (95% CI [71.88%, 94.73%]), and 88.5% (95% CI [77.51%, 99.53%]) in the training set (C) and 89.5% (95% CI [76.93%, 99.99%]), 87.1% (95% CI [76.25%, 97.98%]), and 82.3% (95% CI [67.38%, 97.39%]) in the testing set, respectively (D)

In addition to the RSF model, we also used another supervised machine learning‐based model, the SVM model, to predict the prognosis of HGG. In the SVM model, a 77% prediction accuracy was achieved using a four‐feature combination (Figure 5A). Specifically, age, grade, GTV, and CTV1 were identified as prognostic predictors (Figure 5B). Subsequently, the AUCs for 1‐, 2‐, and 3‐year survival were 82.6% (95% CI [71.95%, 93.21%]), 83.3% (95% CI [71.88%, 94.73%]), and 88.5% (95% CI [77.51%, 99.53%]), respectively, in the training set (Figure 5C) and 89.5% (95% CI [76.93%, 99.99%]), 87.1% (95% CI [76.25%, 97.98%]), and 82.3% (95% CI [67.38%, 97.39%]), respectively, in the testing set (Figure 5D) (Table 2).

### 3.5 Classical analysis of potential prognostic factors

To evaluate the performance of the machine learning‐based models, we also used the classical multivariate CPH model to predict the survival of patients. As shown in Table 3, we found that age (*p* = 0.011), KPS (*p* = 0.017), grade (*p* = 0.017), and D90 (*p* = 0.049) were significantly correlated with OS. Specifically, older age (>60 years), higher grade (Grade IV), and high D90 dose (>5829.90 cGy) were related to poorer survival, with HRs of 2.902, 12.377, and 2.236, respectively, and a higher KPS score (>60) was related to improved survival (HR =0.286). The AUCs for 1‐, 2‐, and 3‐year survival were 83.5% (95% CI [72.36%, 94.66%]), 87.1% (95% CI [76.96%, 97.24%]) and 91.6% (95% CI [82.05%, 99.99%]), respectively, in the training set and 87.9% (95% CI [74.01%, 99.99%]), 87.3% (95% CI [76.04%, 98.68%]), and 84.9% (95% CI [68.93%, 99.99%]), respectively, in the testing set (Figure 5B) (Table 2).

**TABLE 3:** Classical analysis of potential clinical and DVH features influencing OS

Variable	Univariate analysis		Multivariate analysis	
	Hazard ratio (95% CI)	*p*	Hazard ratio (95% CI)	*p*
Age		0.000		0.011
≤60	1		1	
>60	4.551 [2.089, 9.914]		2.902 [1.271, 6.623]	
Grade		0.008		0.017
III	1		1	
IV	15.424 [2.074, 114.720]		12.377 [1.581, 96.917]	
KPS		0.007		0.017
≤60	1		1	
>60	0.276 [0.109, 0.702]		0.286 [0.102, 0.803]	
D90 (cGy)		0.162		0.049
≤5829.90	1		1	
>5829.90	1.742 [0.801, 3.792]		2.236 [1.002, 4.989]	

### 3.6 Assessment of the predictive capabilities of the RSF model

**FIGURE 6:**
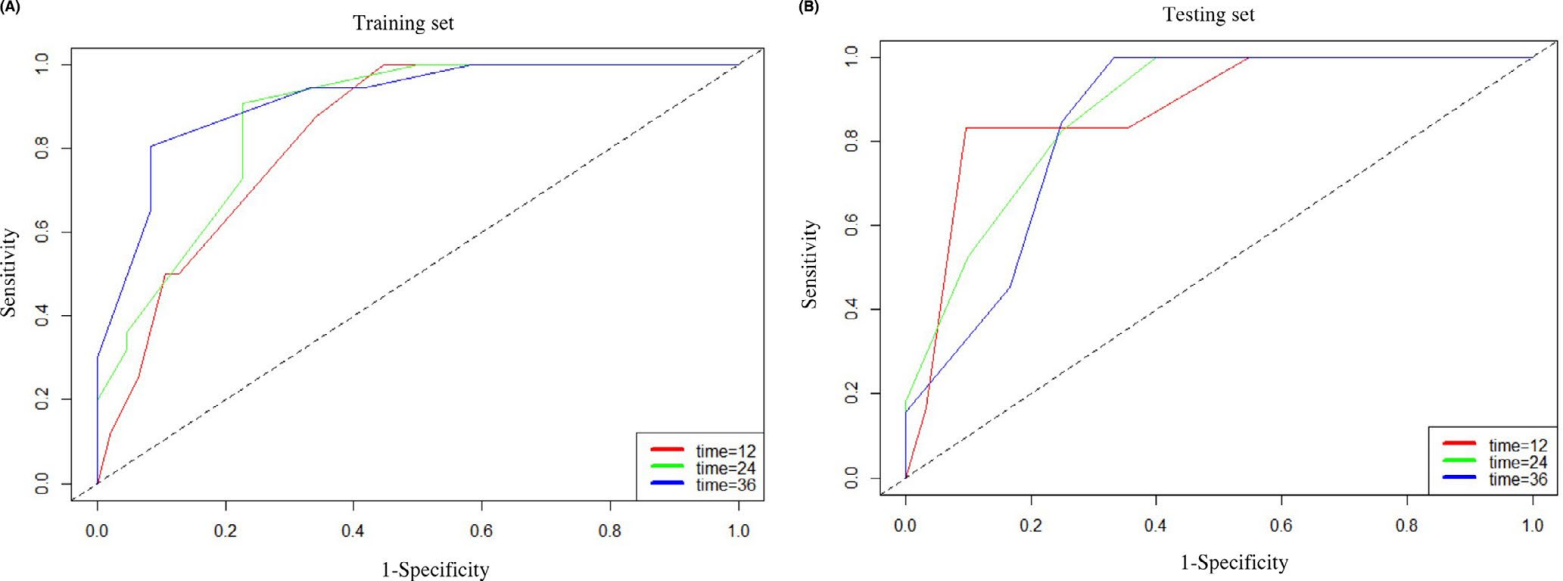
In the CPH model, the AUCs for 1‐, 2‐, and 3‐year survival were 83.5% (95% CI [72.36%, 94.66%]), 87.1% (95% CI [76.96%, 97.24%]), and 91.6% (95% CI [82.05%, 99.99%]), respectively, in the training set (A) and 87.9% (95% CI [74.01%, 99.99%]), 87.3% (95% CI [76.04%, 98.68%]), and 84.9% (95% CI [68.93%, 99.99%]) in the testing set, respectively (B)

The performance of the model was verified by calibration and discrimination. The calibration plots matched well with the ideal 45‐degree line, implying good consistency between the model predictions and actual observations of the 1‐, 2‐, and 3‐year survival probabilities in the training and validation cohorts for the RSF (Figure S1), SVM (Figure S2) and CPH models (Figure S3). In terms of discrimination ability, to compare the performance of the three models, we calculated the c‐index, which measures the concordance between the predicted risks and the actual survival, applied to both the training and testing sets. As shown in Table 4, the c‐indexes of the training and testing sets were 0.824 and 0.847 for the RSF model, 0.792 and 0.823 for the SVM model, and 0.821 and 0.811 for the CPH model, implying the good performance of the RSF model (Figure 6).

**TABLE 4:** C‐index of RSF, SVM, and CPH model

Machine learning‐based RSF model	Training set	0.824
	Testing set	0.847
Machine learning‐based SVM model	Training set	0.792
	Testing set	0.823
Classical CPH model	Training set	0.821
	Testing set	0.811

**FIGURE 7:**
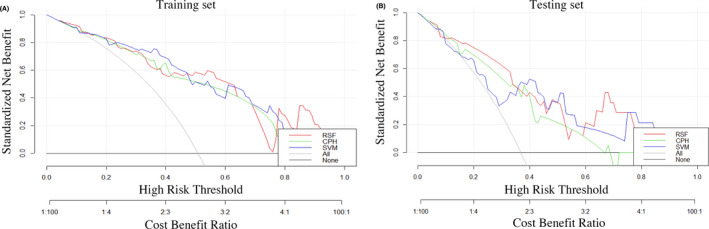
DCA curves for the clinical benefit and the corresponding scope of application of the three models in the training (A) and testing sets (B). The RSF model had greater net benefits than the SVM and CPH models in the testing set

Moreover, DCA calculates the net benefit to evaluate whether a model is clinically useful. The results in Figure 7 show that the RSF model offered the best clinical utility, with greater net benefits than the SVM and CPH models. The results indicated that the RSF model has greater clinical utility than the SVM and CPH models in terms of survival prediction. Overall, the RSF model shows better performance and improvement over the SVM and CPH models.

## 4 DISCUSSION

In this study, we used two machine learning‐based models and a classical model to predict OS by analyzing patient clinical parameters and DVH parameters. The RSF model showed the best performance and improvement among the three models, with c‐indexes of 0.824 and 0.847 for the training and testing sets, respectively, followed by the SVM model (0.792/0.823) and CPH model (0.821/0.811). RSF is one of the widely used methods of machine learning, and it bypasses the requirement of parametric or semiparametric constraints on the underlying distributions and automatically deals with high‐level interactions and higher order terms in variables, to generate much more accurate predictions.^24^ SVM is also a learning method for the classification of linear and nonlinear data. However, it was generally proposed for response prediction and was recently extended to survival prediction.^25^ In our study, the RSF model also outperformed the SVM model in predicting survival. Additionally, the CPH model was not designed to predict outcomes but to infer the impact of variables on a survival curve, while machine learning is more suitable for making predictions. The predictive accuracy of the CPH model as calculated by the c‐index was the lowest among the three models.

In the RSF model, the AUCs were 92.4%, 87.7%, and 84.0% for predicting 1‐, 2‐, and 3‐year survival, respectively, in the testing set, suggesting that the model had good predictive ability. Thus, we can divide patients into high‐ and low‐risk groups to predict OS according to this model. These results demonstrate the possibility of identifying HGG patients who are at risk of having a poor outcome. This stratification of risk will help clinicians provide much more individualized interventions and strategies in cancer therapy. The higher accuracy and better performance of the RSF model make it highly valuable for predicting survival in HGG patients. In fact, a few researchers have expended great effort to develop a prognostic prediction model for this highly malignant cancer, proposing a survival prediction model for HGG based on MRI radiomic features combined with genetic and clinical risk factors.^26^, ^27^, ^28^, ^29^ Imaging features from fluorodeoxyglucose‐positron emission tomography (FDG‐PET) have also been used to predict survival in recurrent HGG.^30^ This field has attracted increasing attention because of its noninvasive nature and easy access.^31^ However, there are challenges in image analyses, including the lack of standardization in imaging acquisition, poor reproducibility, and complex quantitative features.^32^ Hence, comparing results across institutions can be challenging, thereby limiting its clinical use. Therefore, we did not include the imaging features of HGG patients in this study. Instead, we used the DVH features combined with clinicopathological variables to construct a prediction model. Few studies make use of the DVH ‐based treatment plan, which is essential for HGG patients after surgery.

In the RSF model, feature selection identified age, GTV, grade, KPS, IDH, and D99 as the most important predictors of HGG. Among them, age, grade, and KPS are all well‐known prognostic factors and were unsurprisingly selected as significant parameters in the RSF model.^6^ Regarding well‐studied molecular parameters, such as IDH1 R132H mutation, 1p/19q chromosomal codeletion, and MGMT promoter methylation, genomic sequencing, fluorescence in situ hybridization and methylation‐specific PCR are recommended for detection.^7^, ^8^ However, these detection methods can be cost‐prohibitive and are not covered by basic medical insurance in China. Therefore, our oncology center used immunohistochemistry for IDH1 R132H mutation and MGMT expression. The 1p/19q chromosomal codeletion is not routinely detected, so we did not include it in this study. We identified the IDH1 R132H mutation as a significant variable in the RSF model but not in the SVM and CPH models, which indicates that the RSF model might be more appropriate and accurate to some extent. GMT expression was not identified in the three models. This may be because we used immunohistochemistry for its detection, which is neither accurate nor sensitive.

Furthermore, in the RSF model, GTV and D99 were identified as prognostic predictors. These results support the notion that dosimetric parameters can predict patient prognosis. GTV was defined as the surgical resection cavity plus any residual tumor. A higher GTV might indicate a larger preoperative tumor volume. It is difficult to irradiate large tumors with sufficient doses due to the limited toxicity tolerance of adjacent normal tissues. In addition, tumor hypoxia is more pronounced in larger tumors and is associated with treatment failure due to decreased radiosensitivity. D99, also called the near‐minimum absorbed dose, presents the dose that covers 99% of the target volume. The near‐minimum absorbed doses, including D99 and D98, can be used to evaluate the uniformity of dose distribution^33^ and are recommended when reporting treatment plans.^21^, ^34^ D99 is more accurate than the minimum absorbed dose, because the minimum absorbed dose is often located in a high‐gradient region, making it highly sensitive to the dose calculation resolution and the accuracy of target volume delineation. In addition, D99 was a potential predictive parameter in the tumor control probability model and demonstrated a better correlation with clinical outcome.^35^

We did not identify EUD or TCP, which are very important biophysical factors for predicting radiobiological effects and tumor control probability, as significant prognostic predictors in the RSF model. As biological optimization parameters, EUD and TCP are increasingly being applied to treatment plans, but their credibility is still questionable. The model parameters, such as TCD_50_ and γ_50,_ are not constant and can vary according to tumor volume, intrinsic radiosensitivity, tumor heterogeneity, tumor hypoxia, and so on. In addition, the tumor is time varying during treatment, so these parameters might change over time in different regions and to different extents. As a result, it is difficult to predict survival using EUD and TCP models, and the results in this study at least confirmed the limitations and instability of these biophysical models. Tumor heterogeneity and radiobiological factors should be considered in EUD and TCP models in the future.

There are two major strengths of this study. First, there are many reports on the survival analysis of patients with HGG based on clinicopathological factors and data mining of imaging variables. However, only a limited number of studies have investigated the importance of DVH for survival.^36^ In this study, clinical and DVH features were combined to construct a prognostic prediction model, and we found that radiation dose information can affect prognosis. Therefore, DVH parameters should be taken into consideration in future prognostic studies of HGG. Second, we compared the performance of different models in the survival prediction of HGG and demonstrated that the RSF model showed a higher performance with HGG patient data than the SVM and CPH models. The RSF model offers potential benefit to patients by stratifying their risk and guiding clinicians in developing much more individualized interventions and strategies.^12^ Undoubtedly, some limitations also exist in this study. First, the sample size was relatively small, which led to higher AUCs for 1‐ and 2‐year survival in the testing set than in the training set for the SVM and RSF models.^37^ To compare the performance of different models and select the best one, we did not conduct repeated cross‐validation, which is a useful alternative in machine learning for small sample size studies.^38^ Second, this was a single institute‐based study, without multi‐institutional validation. Third, longer follow‐up is needed, especially for Grade III glioma patients. Finally, molecular parameters detected by standard diagnostic methods and radiomics features should be included in future studies.

In summary, we identified age, GTV, grade, KPS, IDH, and D99 as important variables associated with survival in the RSF model. The machine learning‐based RSF model, which integrates both clinical and DVH variables, is an improved and useful tool for predicting the survival of HGG patients. Additional multi‐institutional studies with more patients are needed to assess the interactions between clinical and DVH features and prognosis.

## Ethics approval

This project was approved by the Ethical Committee of the Second Affiliated Hospital of Zhejiang University School of Medicine.

## Consent to participate

Informed consent to participate was obtained from all patients.

## Consent for publication

Informed consent for publication was obtained from all authors.

## CONFLICT OF INTEREST

The authors have no conflict of interest.

## Supporting information

Fig S1

Fig S2

Fig S3

Table S1

## Data Availability

The data that support the findings of this study are available from the corresponding author upon reasonable request.

## References

[1] Ostrom QT , Cote DJ , Ascha M , Kruchko C , Barnholtz‐Sloan JS . Adult glioma incidence and survival by race or ethnicity in the United States from 2000 to 2014. JAMA Oncol. 2018;4(9):1254‐1262. 10.1001/jamaoncol.2018.1789. 29931168 PMC6143018

[2] Ostrom QT , Bauchet L , Davis FG , et al. The epidemiology of glioma in adults: a “state of the science” review. Neuro Oncol. 2014;16(7):896‐913. 10.1093/neuonc/nou087. 24842956 PMC4057143

[3] Louis DN , Perry A , Reifenberger G , et al. The 2016 World Health Organization classification of tumors of the central nervous system: a summary. Acta Neuropathol. 2016;131(6):803‐820. 10.1007/s00401-016-1545-1. 27157931

[4] Stupp R , Brada M , van den Bent MJ , et al. High‐grade glioma: ESMO Clinical Practice Guidelines for diagnosis, treatment and follow‐up. Ann Oncol. 2014;25(suppl_3):iii93‐iii101. 10.1093/annonc/mdu050. 24782454

[5] de Groot JF . High‐grade Gliomas. Contin Lifelong Learn Neurol. 2015;21(2):332‐344. https://journals.lww.com/continuum/Fulltext/2015/04000/High_grade_Gliomas.8.aspx. 10.1212/01.CON.0000464173.58262.d9 25837899

[6] Álvarez de Eulate‐Beramendi S , Álvarez‐Vega MA , Balbin M , Sanchez‐Pitiot A , Vallina‐Alvarez A , Martino‐González J . Prognostic factors and survival study in high‐grade glioma in the elderly. Br J Neurosurg. 2016;30(3):330‐336. 10.3109/02688697.2016.1139049. 26828095

[7] Weller M , Wick W , Aldape K , et al. Glioma. Nat Rev Dis Prim. 2015;1(1):15017. 10.1038/nrdp.2015.17. 27188790

[8] Burgenske DM , Yang J , Decker PA , et al. Molecular profiling of long‐term IDH‐wildtype glioblastoma survivors. Neuro Oncol. 2019;21(11):1458‐1469. 10.1093/neuonc/noz129. 31346613 PMC6827834

[9] Yang K , Tian J , Zhang B , et al. A multidimensional nomogram combining overall stage, dose volume histogram parameters and radiomics to predict progression‐free survival in patients with locoregionally advanced nasopharyngeal carcinoma. Oral Oncol. 2019;98:85‐91. 10.1016/j.oraloncology.2019.09.022. 31569054

[10] Rijkmans EC , Marijnen CAM , van Triest B , et al. Predictive factors for response and toxicity after brachytherapy for rectal cancer; results from the HERBERT study. Radiother Oncol. 2019;133:176‐182. 10.1016/j.radonc.2019.01.034. 30935576

[11] Katzman JL , Shaham U , Cloninger A , Bates J , Jiang T , Kluger Y . DeepSurv: personalized treatment recommender system using a Cox proportional hazards deep neural network. BMC Med Res Methodol. 2018;18(1):24. 10.1186/s12874-018-0482-1. 29482517 PMC5828433

[12] Kim DW , Lee S , Kwon S , Nam W , Cha IH , Kim HJ . Deep learning‐based survival prediction of oral cancer patients. Sci Rep. 2019;9(1):1‐10. 10.1038/s41598-019-43372-7. 31061433 PMC6502856

[13] Wang H , Li G . A selective review on random survival forests for high dimensional data. Quant bio‐science. 2017;36(2):85‐96. 10.22283/qbs.2017.36.2.85. PMC6364686 30740388

[14] Silva A , Oliveira T , Novais P , et al. In: Lindgren H , De Paz JF , Novais P , eds. Developing an Individualized Survival Prediction Model for Colon Cancer BT–Ambient Intelligence–Software and Applications–7th International Symposium on Ambient Intelligence (ISAm I 2016). Cham: Springer International Publishing; 2016:87‐95.

[15] Bhavsar H , Panchal MH . A review on support vector machine for data classification. Int J Adv Res Comput Eng Technol. 2012;1(10).

[16] Jakkula V . Tutorial on support vector machine (svm). Sch EECS: Washingt State Univ; 2006:37.

[17] Serventi J , Behr J . Surgery and evidence‐based treatments in patients with newly diagnosed high‐grade glioma. Semin Oncol Nurs. 2018;34(5):443‐453. 10.1016/j.soncn.2018.10.009. 30409553

[18] Zhou X , Liao X , Zhang B , et al. Recurrence patterns in patients with high‐grade glioma following temozolomide‐based chemoradiotherapy. Mol Clin Oncol. 2016;5(2):289‐294. 10.3892/mco.2016.936. PMC4950878 27446566

[19] Niyazi M , Brada M , Chalmers AJ , et al. ESTRO‐ACROP guideline “target delineation of glioblastomas”. Radiother Oncol. 2016;118(1):35‐42. 10.1016/j.radonc.2015.12.003. 26777122

[20] Taghian A , duBois W , Budach W , Baumann M , Freeman J , Suit H . In vivo radiation sensitivity of glioblastoma multiforme. Int J Radiat Oncol Biol Phys. 1995;32(1):99‐104. 7721644 10.1016/0360-3016(94)00494-6

[21] Holmes T , Das R , Low D , et al. American Society of Radiation Oncology recommendations for documenting intensity‐modulated radiation therapy treatments. Int J Radiat Oncol Biol Phys. 2009;74(5):1311‐1318. 19616738 10.1016/j.ijrobp.2009.04.037

[22] Gay HA , Niemierko A . A free program for calculating EUD‐based NTCP and TCP in external beam radiotherapy. Phys Medica. 2007;23(3):115‐125. 10.1016/j.ejmp.2007.07.001. 17825595

[23] Okunieff P , Morgan D , Niemierko A , Suit HD . Radiation dose‐response of human tumors. Int J Radiat Oncol Biol Phys. 1995;32(4):1227‐1237. 7607946 10.1016/0360-3016(94)00475-z

[24] Mogensen UB , Ishwaran H , Gerds TA . Evaluating random forests for survival analysis using prediction error curves. J Stat Softw. 2012;50(11):1‐23. 10.18637/jss.v050.i11. 25317082 PMC4194196

[25] Lee S , Lim H . Review of statistical methods for survival analysis using genomic data. Genomics Inf. 2019;17(4):e41. 10.5808/GI.2019.17.4.e41. PMC6944043 31896241

[26] Tan Y , Mu W , Wang X , Yang G , Gillies RJ , Zhang H . Improving survival prediction of high‐grade glioma via machine learning techniques based on MRI radiomic, genetic and clinical risk factors. Eur J Radiol. 2019;120:108609. 10.1016/j.ejrad.2019.07.010. 31606714

[27] Zhang J , Jiang J , Zhao L , et al. Survival prediction of high‐grade glioma patients with diffusion kurtosis imaging. Am J Transl Res. 2019;11(6):3680. 31312379 PMC6614625

[28] Garzín B , Emblem KE , Mouridsen K , et al. Multiparametric analysis of magnetic resonance images for glioma grading and patient survival time prediction. Acta radiol. 2011;52(9):1052‐1060. 10.1258/AR.2011.100510. 21969702

[29] Shboul ZA , Alam M , Vidyaratne L , Pei L , Elbakary MI , Iftekharuddin KM . Feature‐guided deep radiomics for glioblastoma patient survival prediction. Front Neurosci. 2019;13:966. 10.3389/fnins.2019.00966. 31619949 PMC6763591

[30] Colavolpe C , Chinot O , Metellus P , et al. FDG‐PET predicts survival in recurrent high‐grade gliomas treated with bevacizumab and irinotecan. Neuro Oncol. 2012;14(5):649‐657. 10.1093/neuonc/nos012. 22379188 PMC3337300

[31] Jeong J , Ali A , Liu T , Mao H , Curran WJ , Yang X . Radiomics in Cancer Radiotherapy: a Review. arXiv Prepr arXiv191002102. 2019.

[32] Rizzo S , Botta F , Raimondi S , et al. Radiomics: the facts and the challenges of image analysis. Eur Radiol Exp. 2018;2(1):1‐8. 30426318 10.1186/s41747-018-0068-z PMC6234198

[33] Gregoire V , MacKie TR . Dose prescription, reporting and recording in intensity‐modulated radiation therapy: a digest of the ICRU report 83. Imaging Med. 2011;3(3):367‐373. 10.2217/iim.11.22. 21802333

[34] Hodapp N . The ICRU Report 83: prescribing, recording and reporting photon‐beam intensity‐modulated radiation therapy (IMRT). Strahlenther Onkol. 2012;188(1):97‐99. 10.1007/s00066-011-0015-x. 22234506

[35] Sood SS , Pokhrel D , Badkul R , et al. Correlation of clinical outcome, radiobiological modeling of tumor control, normal tissue complication probability in lung cancer patients treated with SBRT using Monte Carlo calculation algorithm. J Appl Clin Med Phys. 2020;21(10):56‐62. 10.1002/acm2.13004. PMC7592969 32794632

[36] Mizutani T , Magome T , Igaki H , et al. Optimization of treatment strategy by using a machine learning model to predict survival time of patients with malignant glioma after radiotherapy. J Radiat Res. 2019;60(6):818‐824. 10.1093/jrr/rrz066. 31665445 PMC7357235

[37] Schutten M , Wiering MA . An analysis on better testing than training performances on the iris dataset. Belgian Dutch Artif Intell Conf. 2016;8:10‐11. https://www.rug.nl/research/portal/en/publications/an‐analysis‐on‐better‐testing‐than‐training‐performances‐on‐the‐iris‐dataset(bf47f40f‐8e2d‐4f60‐a65c‐414d2f80ae5c).html.

[38] Schaffer C . Technical note: selecting a classification method by cross‐validation. Mach Learn. 1993;13(1):135‐143. 10.1023/A:1022639714137.

