# Supplementary figures and images for: A machine learning‐based survival prediction model of high grade glioma by integration of clinical and dose‐volume histogram parameters
Source: Cancer Med. 2021 Mar 24;10(8):2774–86. doi: 10.1002/cam4.3838 (PMC8026951; doi:10.1002/cam4.3838)

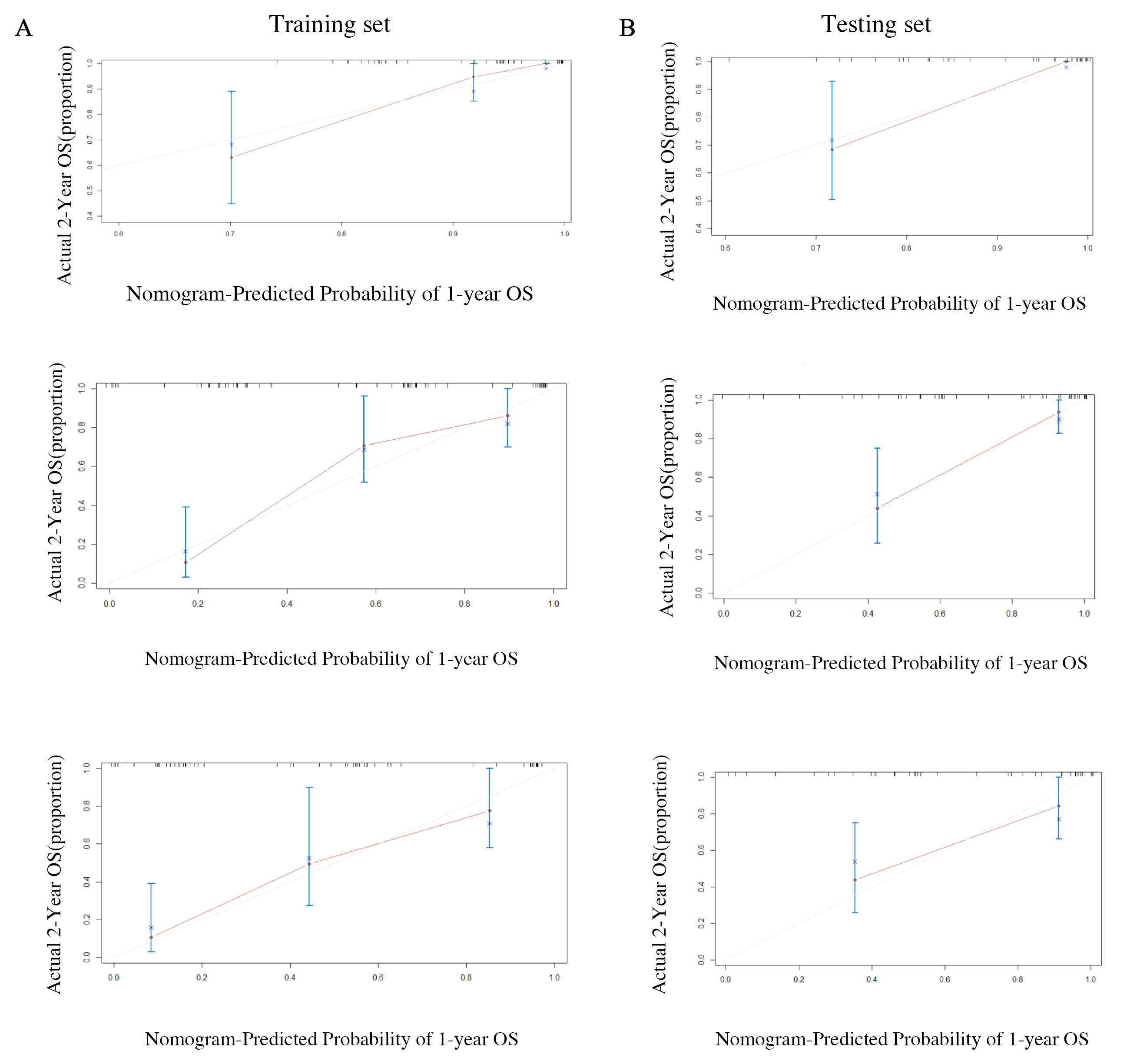

Supplement: Supplementary file 1 — Fig S1 [file CAM4-10-2774-s002.tif]

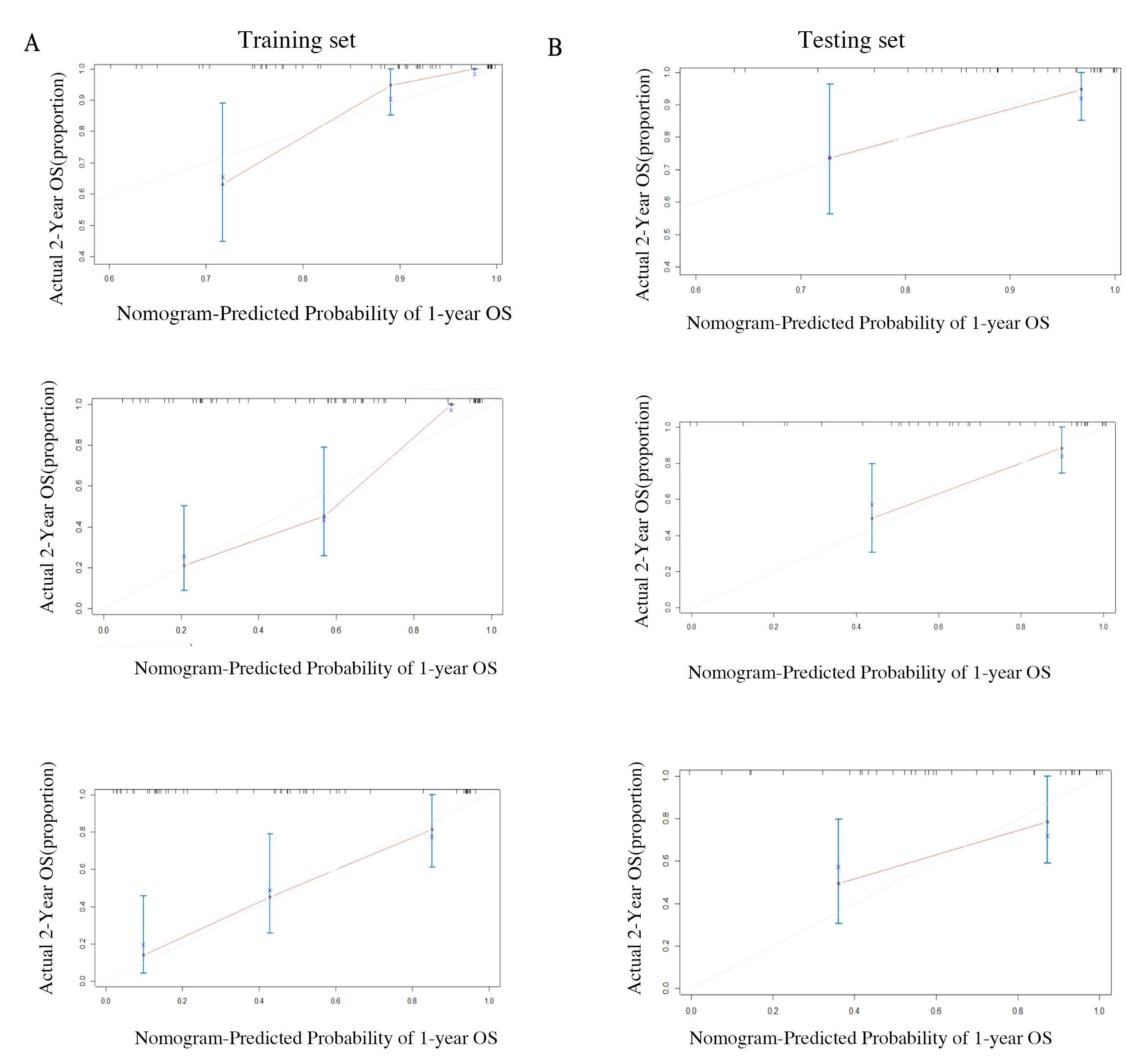

Supplement: Supplementary file 2 — Fig S2 [file CAM4-10-2774-s001.tif]

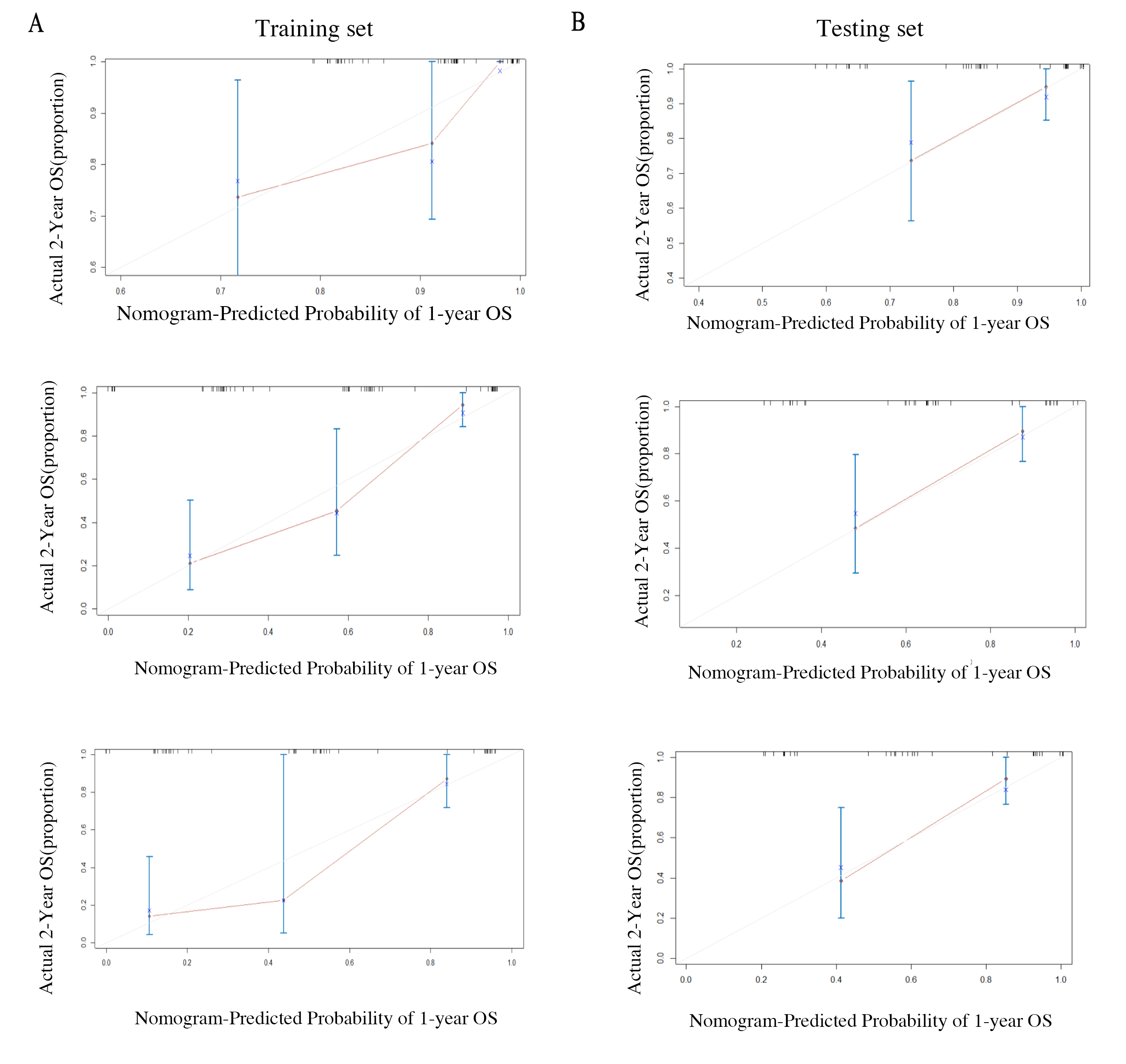

Supplement: Supplementary file 3 — Fig S3 [file CAM4-10-2774-s003.tif]
