# Supplementary material for: A machine learning‐based survival prediction model of high grade glioma by integration of clinical and dose‐volume histogram parameters
Source: Cancer Med. 2021 Mar 24;10(8):2774–86. doi: 10.1002/cam4.3838 (PMC8026951; doi:10.1002/cam4.3838)
Supplement: Supplementary file 4 — Table S1 [file CAM4-10-2774-s004.docx]

| **Supplementary Table 1. DVH features of included patients** | | | | | | |
| --- | --- | --- | --- | --- | --- | --- |
|  | **Total Patients** | | **Training Set** | | **Testing Set** | |
| **Dosimetric parameters** | N | % | N | % | N | % |
| **GTV(cm3)** |  |  |  |  |  |  |
| ≤104.48 | 13 | 13.68% | 8 | 14.04% | 5 | 13.16% |
| >104.48 | 82 | 86.32% | 49 | 85.96% | 33 | 86.84% |
| **CTV1(cm3)** |  |  |  |  |  |  |
| ≤226.91 | 11 | 11.58% | 6 | 10.53% | 5 | 13.16% |
| >226.91 | 84 | 88.42% | 51 | 89.47% | 33 | 86.84% |
| **CTV2(cm3)** |  |  |  |  |  |  |
| ≤348.18 | 12 | 12.63% | 7 | 12.28% | 5 | 13.16% |
| >348.18 | 83 | 87.37% | 50 | 87.72% | 33 | 86.84% |
| **CTV2 Equiv_Sphere_Diam(cm)** | |  |  |  |  |  |
| ≤8.72 | 12 | 12.63% | 7 | 12.28% | 5 | 13.16% |
| >8.72 | 83 | 87.37% | 50 | 87.72% | 33 | 86.84% |
| **CTV2 Min_Dose(cGy)** |  |  |  |  |  |  |
| ≤4710.00 | 45 | 47.37% | 25 | 43.86% | 20 | 52.63% |
| >4710.00 | 50 | 52.63% | 32 | 56.14% | 18 | 47.37% |
| **CTV2 Max_Dose(cGy)** |  |  |  |  |  |  |
| ≤6320.00 | 84 | 88.42% | 50 | 87.72% | 34 | 89.47% |
| >6320.00 | 11 | 11.58% | 7 | 12.28% | 4 | 10.53% |
| **CTV2 Mean_Dose(cGy)** |  |  |  |  |  |  |
| ≤5904.30 | 83 | 87.37% | 50 | 87.72% | 33 | 86.84% |
| >5904.30 | 12 | 12.63% | 7 | 12.28% | 5 | 13.16% |
| **CTV2 Modal_Dose(cGy)** |  |  |  |  |  |  |
| ≤5831.71 | 59 | 62.11% | 35 | 61.40% | 24 | 63.16% |
| >5831.71 | 36 | 37.89% | 22 | 38.60% | 14 | 36.84% |
| **CTV2 Median_Dose(cGy)** |  |  |  |  |  |  |
| ≤5961.27 | 85 | 89.47% | 51 | 89.47% | 34 | 89.47% |
| >5961.27 | 10 | 10.53% | 6 | 10.53% | 4 | 10.53% |
| **CTV2 STD(cGy)** |  |  |  |  |  |  |
| ≤326.70 | 19 | 20.00% | 11 | 19.30% | 8 | 21.05% |
| >326.70 | 76 | 80.00% | 46 | 80.70% | 30 | 78.95% |
| **CTV2 TCP(%)** |  |  |  |  |  |  |
| ≤54.14 | 24 | 54.39% | 10 | 17.54% | 14 | 36.84% |
| >54.14 | 71 | 145.61% | 47 | 82.46% | 24 | 63.16% |
| **CTV2 EUD(cGy)** |  |  |  |  |  |  |
| ≤61.26 | 24 | 25.26% | 10 | 17.54% | 14 | 36.84% |
| >61.26 | 71 | 74.74% | 47 | 82.46% | 24 | 63.16% |
| **CTV2 D99(cGy)** |  |  |  |  |  |  |
| ≤4901.89 | 86 | 90.53% | 51 | 89.47% | 35 | 92.11% |
| >4901.89 | 9 | 9.47% | 6 | 10.53% | 3 | 7.89% |
| **CTV2 D98(cGy)** |  |  |  |  |  |  |
| ≤5047.00 | 85 | 89.47% | 49 | 85.96% | 36 | 94.74% |
| >5047 | 10 | 10.53% | 8 | 14.04% | 2 | 5.26% |
| **CTV2 D95(cGy)** |  |  |  |  |  |  |
| ≤5557.00 | 61 | 64.21% | 37 | 64.91% | 24 | 63.16% |
| >5557.00 | 34 | 35.79% | 20 | 35.09% | 14 | 36.84% |
| **CTV2 D90(cGy)** |  |  |  |  |  |  |
| ≤5829.90 | 34 | 35.79% | 16 | 28.07% | 18 | 47.37% |
| >5829.90 | 61 | 64.21% | 41 | 71.93% | 20 | 52.63% |
| **CTV2 D85(cGy)** |  |  |  |  |  |  |
| ≤5563.00 | 88 | 92.63% | 51 | 89.47% | 37 | 97.37% |
| >5563.00 | 7 | 7.37% | 6 | 10.53% | 1 | 2.63% |
| **CTV2 D80(cGy)** |  |  |  |  |  |  |
| ≤6048.95 | 25 | 26.32% | 13 | 22.81% | 12 | 31.58% |
| >6048.95 | 70 | 73.68% | 44 | 77.19% | 26 | 68.42% |
| **CTV2 D75(cGy)** |  |  |  |  |  |  |
| ≤6069.00 | 28 | 29.47% | 13 | 22.81% | 15 | 39.47% |
| >6069.00 | 67 | 70.53% | 44 | 77.19% | 23 | 60.53% |
| **CTV2 D70(cGy)** |  |  |  |  |  |  |
| ≤5855.45 | 79 | 83.16% | 48 | 84.21% | 31 | 81.58% |
| >5855.45 | 16 | 16.84% | 9 | 15.79% | 7 | 18.42% |
| **CTV2 D65(cGy)** |  |  |  |  |  |  |
| ≤5886.01 | 80 | 84.21% | 49 | 85.96% | 31 | 81.58% |
| >5886.01 | 15 | 15.79% | 8 | 14.04% | 7 | 18.42% |
| **CTV2 D60(cGy)** |  |  |  |  |  |  |
| ≤5915.00 | 84 | 88.42% | 51 | 89.47% | 33 | 86.84% |
| >5915.00 | 11 | 11.58% | 6 | 10.53% | 5 | 13.16% |
| **CTV2 D55(cGy)** |  |  |  |  |  |  |
| ≤5940.51 | 85 | 89.47% | 51 | 89.47% | 34 | 89.47% |
| >5940.51 | 10 | 10.53% | 6 | 10.53% | 4 | 10.53% |
| **CTV2 D50(cGy)** |  |  |  |  |  |  |
| ≤5962.55 | 85 | 89.47% | 51 | 89.47% | 34 | 89.47% |
| >5962.55 | 10 | 10.53% | 6 | 10.53% | 4 | 10.53% |
| **CTV2 D45(cGy)** |  |  |  |  |  |  |
| ≤6051.69 | 81 | 85.26% | 47 | 82.46% | 34 | 89.47% |
| >6051.69 | 14 | 14.74% | 10 | 17.54% | 4 | 10.53% |
| **CTV2 D40(cGy)** |  |  |  |  |  |  |
| ≤6106.00 | 78 | 82.11% | 46 | 80.70% | 32 | 84.21% |
| >6106.00 | 17 | 17.89% | 11 | 19.30% | 6 | 15.79% |
| **CTV2 D35(cGy)** |  |  |  |  |  |  |
| ≤6151.00 | 74 | 77.89% | 43 | 75.44% | 31 | 81.58% |
| >6151.00 | 21 | 22.11% | 14 | 24.56% | 7 | 18.42% |
| **CTV2 D30(cGy)** |  |  |  |  |  |  |
| ≤6146.00 | 78 | 82.11% | 46 | 80.70% | 32 | 84.21% |
| >6146.00 | 17 | 17.89% | 11 | 19.30% | 6 | 15.79% |
| **CTV2 D25(cGy)** |  |  |  |  |  |  |
| ≤6157.00 | 79 | 83.16% | 47 | 82.46% | 32 | 84.21% |
| >6157.00 | 16 | 16.84% | 10 | 17.54% | 6 | 15.79% |
| **CTV2 D20(cGy)** |  |  |  |  |  |  |
| ≤6173.19 | 80 | 84.21% | 47 | 82.46% | 33 | 86.84% |
| >6173.19 | 15 | 15.79% | 10 | 17.54% | 5 | 13.16% |
| **CTV2 D15(cGy)** |  |  |  |  |  |  |
| ≤6193.59 | 80 | 84.21% | 47 | 82.46% | 33 | 86.84% |
| >6193.59 | 15 | 15.79% | 10 | 17.54% | 5 | 13.16% |
| **CTV2 D10(cGy)** |  |  |  |  |  |  |
| ≤6216.55 | 81 | 85.26% | 47 | 82.46% | 34 | 89.47% |
| >6216.55 | 14 | 14.74% | 10 | 17.54% | 4 | 10.53% |
| **CTV2 D5(cGy)** |  |  |  |  |  |  |
| ≤6197.98 | 86 | 90.53% | 51 | 89.47% | 35 | 92.11% |
| >6197.98 | 9 | 9.47% | 6 | 10.53% | 3 | 7.89% |
| **CTV2 D2(cGy)** |  |  |  |  |  |  |
| ≤6217.43 | 86 | 90.53% | 51 | 89.47% | 35 | 92.11% |
| >6217.43 | 9 | 9.47% | 6 | 10.53% | 3 | 7.89% |
| **CTV2 D1(cGy）** |  |  |  |  |  |  |
| ≤6229.35 | 87 | 91.58% | 51 | 89.47% | 36 | 94.74% |
| >6229.35 | 8 | 8.42% | 6 | 10.53% | 2 | 5.26% |
| **CTV2 V65(%）** |  |  |  |  |  |  |
| ≤0.03 | 35 | 36.84% | 22 | 38.60% | 13 | 34.21% |
| >0.03 | 60 | 63.16% | 35 | 61.40% | 25 | 65.79% |
| **CTV2 V60(%）** |  |  |  |  |  |  |
| ≤53.0 | 14 | 14.74% | 9 | 15.79% | 5 | 13.16% |
| >53.0 | 81 | 85.26% | 48 | 84.21% | 33 | 86.84% |
| **CTV2 V55(%）** |  |  |  |  |  |  |
| ≤84.6 | 6 | 6.32% | 5 | 8.77% | 1 | 2.63% |
| >84.6 | 89 | 93.68% | 52 | 91.23% | 37 | 97.37% |
| **CTV2 V50(%）** |  |  |  |  |  |  |
| ≤98.9 | 12 | 12.63% | 8 | 14.04% | 4 | 10.53% |
| >98.9 | 83 | 87.37% | 49 | 85.96% | 34 | 89.47% |
